# Case Report: Malignant PEComa of the thyroid combined with follicular carcinoma: a rare case of collision tumor

**DOI:** 10.3389/fonc.2025.1733442

**Published:** 2026-01-02

**Authors:** Yongchen Liu, Yuanpei Lin, Xiaomei Li, Jian Chen, Xinmei Chen, Jiangbo Deng, Zeyu Wu

**Affiliations:** 1 Department of Thyroid Hernia Surgery, Guangdong Provincial People’s Hospital (Guangdong Academy of Medical Sciences), Southern Medical University, Guangzhou, Guangdong, China; 2 Department of Cervical Thoracic Surgery, People’s Hospital of Yingde City, Yingde, Guangdong, China; 3 Department of Pathology, People’s Hospital of Yingde City, Yingde, Guangdong, China

**Keywords:** perivascular epithelioid cell tumor (PEComa), thyroid follicular carcinoma, collision tumor, immunohistochemistry, pathological diagnosis

## Abstract

We present a rare case of a collision tumor comprising a malignant perivascular epithelioid cell tumor (PEComa) of the thyroid combined with a tiny invasive follicular carcinoma (FTC) in a 72-year-old female patient. The patient presented to the hospital with a painless anterior cervical mass discovered one month prior, and examination revealed a 50 mm × 30 mm mass in the left lobe of the thyroid gland. Ultrasound showed multiple solid and mixed lesions with gross calcifications in both thyroid lobes. The patient underwent total resection of the left thyroid and isthmus plus right subtotal resection. Histopathological examination revealed two distinct tumor components in the left lobe: immunohistochemistry revealed a PEComa component (HMB45+/SMA+/Desmin+, Ki-67 about 50%, P53 missense mutation) and a follicular carcinoma component (TTF-1+/PAX8+/TPO+, Ki-67 about 3% and P53 wild-type). The two components were well demarcated without mutual migration, meeting the diagnostic criteria for collision tumors. This case represents the first reported collision tumor combining thyroid PEComa and FTC, with immunohistochemistry confirming the independent origins of both tumors. The findings expand our understanding of thyroid tumor varieties and highlight the crucial role of Ultrasound and histopathology in diagnosing and managing complex thyroid tumors, serving as a valuable reference for similar cases.

## Introduction

Thyroid tumors are common endocrine system malignancies. Their pathologic classification of thyroid tumors has been continually refined with the advancement of diagnostic techniques. Beyond classical types like papillary and follicular carcinoma, some rare tumors with special differentiation characteristics have been gradually recognized in recent years. Among them, perivascular epithelioid cell tumor (PEComa), as a mesenchymal origin tumor expressing both melanocyte and smooth muscle markers, has been reported in thyroid primary tumors in an extremely limited manner. According to the available literature, case reports of thyroid PEComa are rare worldwide (1, 2), and its clinicopathological features remain to be further elucidated.

More notably, the phenomenon of “collision tumor”, in which multiple primary tumors coexist in the same organ, has not been systematically studied in the thyroid gland. Collision tumor is a special pathological phenomenon in which two histologically different tumors coexist independently in the same anatomical site without mutual migration. Current reports of thyroid collision tumors have focused on the coexistence of papillary and follicular thyroid carcinomas (3), whereas there has been no clear documentation of collision tumors between different types of malignant tumors, especially between PEComa and tumors of epithelial origin.

In the present study, we report an exceptional case of an elderly female patient with coexisting malignant PEComa and follicular thyroid carcinoma (FTC) in the left lobe of the thyroid gland, which was confirmed to be a typical collision tumor by comprehensive pathologic testing. By systematic immunohistochemical analysis, we demonstrated for the first time the independent coexistence of these two tumors of different origins in the thyroid gland. The reporting of this case will help expand the understanding of the disease spectrum of thyroid tumors and provide a valuable reference for the clinical management of similar cases.

## Case presentation

**Figure 1:**
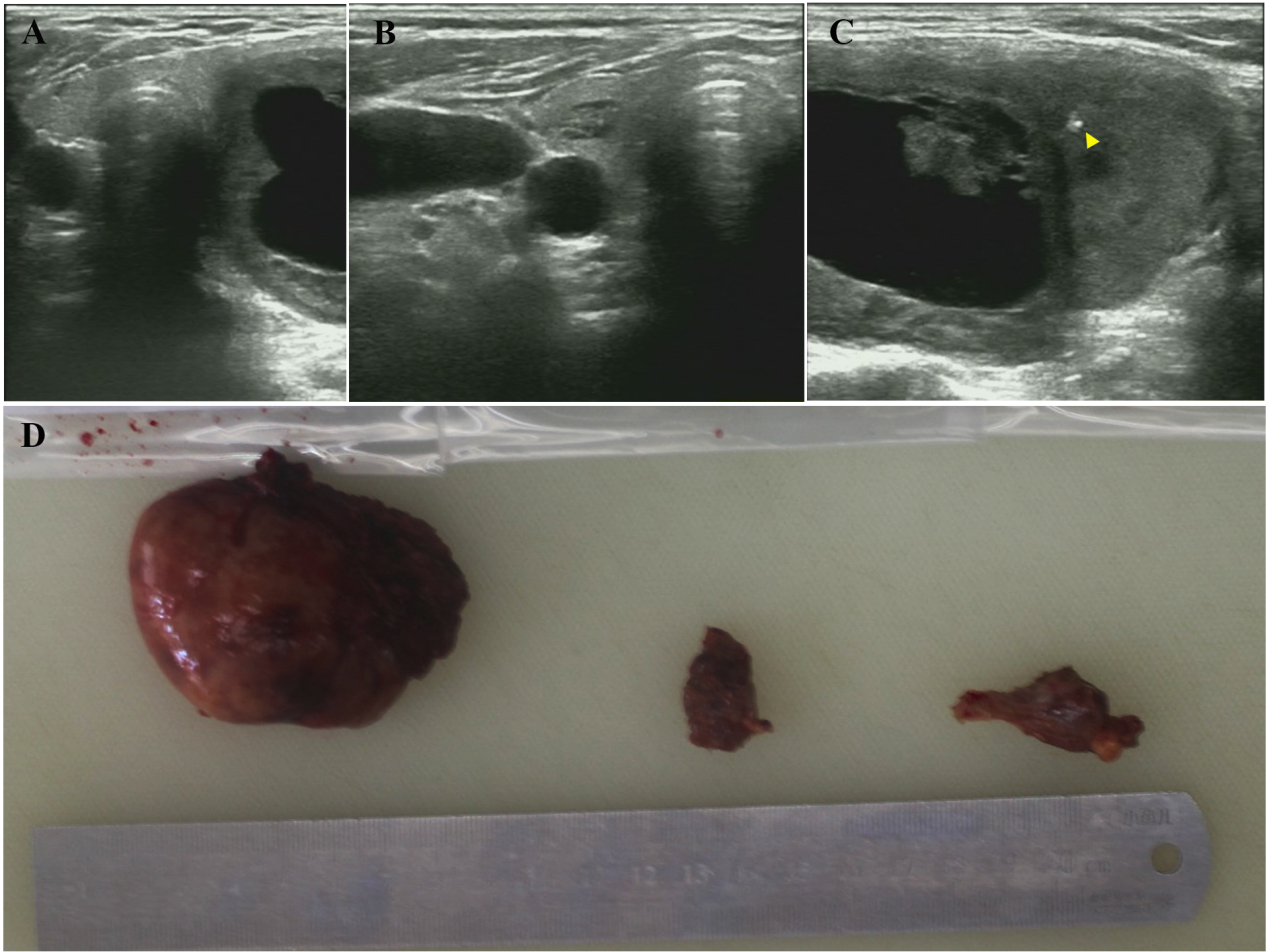
Preoperative ultrasound and gross appearance of the thyroid collision tumor. **(A)** Transverse ultrasound view of the thyroid gland demonstrating bilateral lobes and the isthmus. **(B)** A well-defined, isoechoic nodule (9 mm × 5 mm) in the right thyroid lobe. **(C)** A large, mixed solid-cystic nodule (46 mm × 28 mm) in the left thyroid lobe. The lesion shows irregular anechoic areas (cystic degeneration) and a hyperechoic focus (yellow triangle), suggestive of gross calcification. **(D)** Gross photograph of the resected specimens: left thyroid lobe, partial right thyroid lobe, and thyroid isthmus (from left to right).

The patient, a 72-year-old woman, was admitted with the main complaint of a painless anterior cervical mass that had been found for one month. A month ago, the patient unintentionally found a swelling in the neck, about the size of a walnut, accompanied by mild dysphagia, with no accompanying symptoms such as redness, swelling, pain, palpitations, hand tremor. There were no family history of thyroid disease and no special hobbies. Specialized examination showed that the left lobe of the thyroid gland was enlarged to the second degree, with a palpable swelling of about 50 mm × 30 mm, medium texture, smooth surface, clear borders, good mobility, no tenderness and skin adhesions. Relevant examinations were conducted, including the three routine tests, coagulation tests, liver and kidney function tests, preoperative blood tests, and electrocardiogram. No significant abnormalities were found. The thyroid function test results showed TT4 163.6 nmol/L (reference range 70-148), FT4 22.1 nmol/L (reference range 12-20.2), TSH 0.370 mIU/L (reference range 0.6-4.8), with TT3 and FT3 being normal. The results of TRAb, TPOAb, and TgAb were also normal. Thyroid ultrasonography showed that the size of the left lobe of the thyroid gland was about 61 mm × 45 mm × 34 mm, and the right lobe was about 51 mm × 12 mm × 13 mm, with the isthmus thickness of about 2 mm. multiple isoechoic masses could be seen in the bilateral lobes, with the largest in the left lobe being about 46 mm × 28 mm, and in the right lobe about 9 mm × 5 mm, with clear boundaries and regular morphology. The border was clear, the morphology was regular, the internal echogenicity was uneven, and irregular liquid dark areas and strong echogenic spots with acoustic shadows could be seen. Color Doppler flow imaging (CDFI) showed that blood flow signals could be seen around and inside the lesion. Imaging diagnosis: Multiple solid and mixed occupying lesions in bilateral lobes of the thyroid gland with gross calcification (nodular goiter with cystic degeneration was considered) (**Figures 1A–C**).

The patient’s left thyroid nodule affected appearance, presented with compression symptoms, and was accompanied by mild hyperthyroidism, having surgical indications. This condition needs to be differentiated from nodular goiter, thyroid carcinoma, thyroid adenoma, thyroid intracystic hemorrhage, etc. After fully understanding the condition, the patient and his family members wished to proceed directly to surgery and refused fine-needle aspiration biopsy. Based on the above analysis, we opted for a total resection of the left thyroid gland and isthmus plus partial resection of the right thyroid gland, which can simultaneously meet the resection range requirements for both nodular goiter with hyperthyroidism and thyroid malignancy. In addition, we utilize intraoperative rapid frozen pathological examination to ensure the timely detection of highly malignant pathological types.

**Figure 2:**
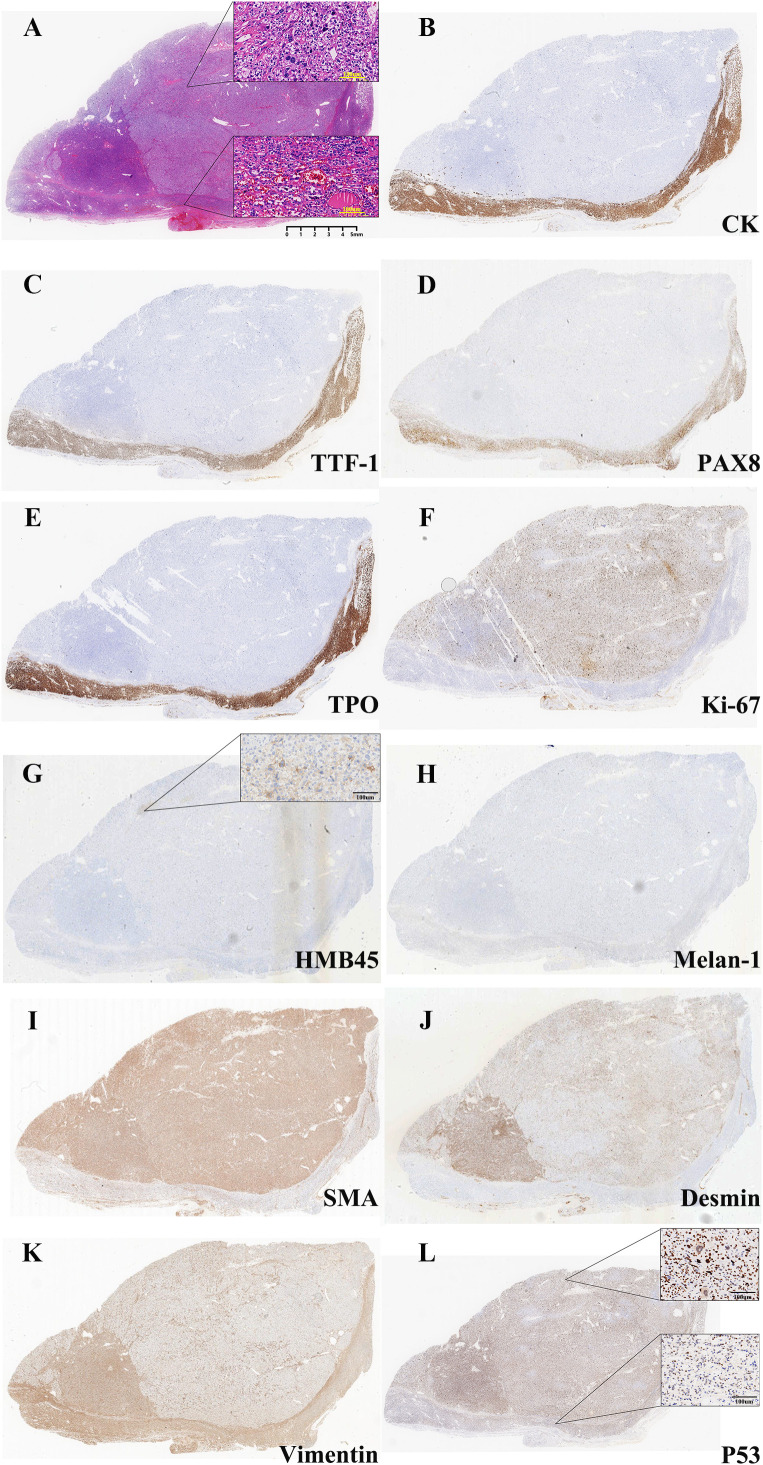
Histopathological and immunohistochemical features of the collision tumor. **(A)** Hematoxylin and eosin (H&E) staining: in PEComa, the tumor nuclei are round, oval, spindle and singular, with coarse chromatin and rich or translucent cytoplasm, which are arranged in islands and sheets. The local areas are coagulated and necrotic (upper), while thyroid follicular hyperplasia in local area invaded capsule and blood vessels in FTC tissue(lower). (Original magnification ×5, inset: ×200). **(B)** Cytokeratin (CK) immunostain is strongly positive in the follicular carcinoma (FTC) component (lower) and completely negative in the PEComa component (upper). (Original magnification ×5). **(C)** Thyroid transcription factor-1 (TTF-1) immunostain shows strong nuclear positivity exclusively in the FTC component. (Original magnification ×5). **(D)** PAX8 immunostain demonstrates nuclear expression in the FTC component, confirming its thyroid follicular cell origin. (Original magnification ×5). **(E)** Thyroid peroxidase (TPO) immunostain is positive in the FTC component. (Original magnification ×5). **(F)** Ki-67 immunostain reveals a high proliferative index (approximately 50%) in the PEComa component (upper), in stark contrast to the low index (approximately 3%) in the FTC component (lower). (Original magnification ×5). **(G)** HMB45 immunostain shows focal but definitive cytoplasmic positivity in the upper PEComa component, supporting the diagnosis. The lower FTC is negative. (Original magnification ×5, inset: ×200). **(H)** Melan-A immunostain is negative in both the upper PEComa and lower FTC components, illustrating an atypical immunophenotype for PEComa. (Original magnification ×5). **(I)** Smooth muscle actin (SMA) immunostain displays diffuse cytoplasmic positivity in the upper PEComa component. The lower FTC is negative. (Original magnification ×5). **(J)** Desmin immunostain is positive in the upper PEComa component, confirming myoid differentiation. (Original magnification ×5). **(K)** Vimentin immunostain is positive in the upper PEComa component, consistent with its mesenchymal lineage. The lower FTC also shows focal positivity. (Original magnification ×5). **(L)** P53 immunostain exhibits a diffuse, strong nuclear staining pattern (“mutant-type”) in the upper PEComa component. In contrast, the lower FTC component shows a wild-type staining pattern with scattered, weak to moderate positivity. (Original magnification ×5, inset: ×200).

After completing the preoperative evaluation, the patient underwent a total resection of the left thyroid gland and isthmus plus partial resection of the right thyroid gland under general anesthesia. Intraoperative exploration showed 6.5cm×6cm×2.5cm of thyroid tissue in the left lobe, and a 6cm×4.5cm grayish-yellow nodule with cystic-solid changes and intact capsule was seen on the cut surface; the tissue of the right lobe and isthmus did not show any obvious abnormality (**Figure 1D**). Intraoperative frozen section diagnosis: (Left lobe of thyroid) nodular goiter, with multiple large, deeply stained or irregular cells scattered within a local nodule. The lesion is considered to be an atypical adenoma. Further diagnosis is pending with routine paraffin section. (Right lobe of thyroid, isthmus) nodular goiter. Postoperative pathological findings: left lobe thyroid: malignant perivascular epithelioid cell tumor (PEComa) Components: immunohistochemistry showed Vimentin (partially +), Smooth muscle actin (SMA) (+), Desmin (+), HMB45 (small foci +), P53 (missense mutation +), and Ki-67 (about 50% +); Follicular carcinoma component: immunohistochemistry showed cytokeratin (CK) (+), PAX8 (+), thyroid transcription factor 1 (TTF1) (+), thyroid peroxidase (TPO) (+), P53 (wild-type +), and Ki-67 (approximately 3%+) (**Figures 2A–L**). The two tumor components were well demarcated without migratory transition, consistent with the diagnosis of Collision tumor (CT). Right lobe thyroid and isthmus: nodular goiter. The patient recovered well in the six-month follow-up after the operation, and there were no complications such as hoarseness and choking on drinking water.

## Discussion

PEComa is a mesenchymal origin tumor with unique immunophenotypic features, and its typical pathological features are the expression of both melanocyte markers (HMB-45, Melan-A) and smooth muscle markers (SMA, Desmin) (4). The tumor is clinically rare and most show benign biological behavior, but malignant cases have been reported (5). PEComas commonly occur in the kidney, liver, uterus, and retroperitoneum, whereas primary PEComa of the thyroid is extremely rare. Although most PEComa present with an inert clinical course, vigilance for malignant potential remains essential.

FTC is a malignant tumor originating from the follicular epithelium of the thyroid gland and accounts for approximately 4% of all thyroid cancers. Compared with papillary thyroid carcinoma, FTC is more aggressive and prone to hematogenous metastasis (commonly to the lungs and bones). Standard treatment options include total thyroidectomy combined with radioactive iodine therapy, which can achieve a 5-year survival rate of more than 90%, with a significantly worse prognosis for patients with advanced disease (6).

A collision tumor is defined by the adjacent coexistence of two or more distinct histologic types of tumors in the same anatomical site, without any mixing or transitional zone between them. This distinguishes it from related concepts such as mixed tumors and composite tumors (7). Although rare, PEComas have been reported to participate in collision tumors with other neoplasms, including endometrial carcinoma and synchronous leiomyomas (8–10). However, a collision between a thyroid PEComa and a follicular thyroid carcinoma (FTC) represents a novel finding, and its mechanism of occurrence and clinical significance remain to be elucidated.

The diagnosis of PEComa is mainly based on histomorphologic and immunohistochemical features. Criteria for malignancy include tumor size >5 cm, invasive growth, high nuclear grade, high mitotic rate (>1/50 HPF), necrosis and vascular invasion (5). Immunohistochemistry is typically characterized by expressions of melanin markers (HMB45, Melan-A), myogenic markers (SMA, Desmin), and negative epithelial markers (CK, TTF-1) (11–14). In this case, immunohistochemistry was critical for distinguishing the two tumor populations. In PEComa, the tumor nuclei are round, oval, spindle and singular, with coarse chromatin and rich or translucent cytoplasm, which are arranged in islands and sheets. The mitosis is like about 5/50 high-power microscopic fields, and local areas are coagulated and necrotic, while thyroid follicular hyperplasia in local area invaded capsule and blood vessels in FTC tissue. The PEComa component demonstrated a classic mesenchymal immunophenotype, which contrasted with the follicular epithelial profile of the FTC component. This immunophenotypic segregation, without crossover of key markers, confirmed the diagnosis of two independent neoplasms. Malignancy in the PEComa was evidenced by a high Ki-67 index (approximately 50%) and a P53 missense mutation pattern, which satisfied the criteria for malignant PEComa. In contrast, the FTC component showed only tiny infiltrative foci, a low Ki-67 index (approximately 3%), and a wild-type P53 pattern, consistent with a low-grade malignancy. Histologically, the two components were well-demarcated with no transitional zone. Their immunophenotypes were distinct and non-overlapping, providing conclusive evidence for their independent origins and establishing the diagnosis of a collision tumor.

The treatment of malignant PEComa is based on complete surgical resection. For inoperable or metastatic cases, targeted therapy with mTOR inhibitors (e.g., sirolimus) may be attempted, but the efficacy of the treatment is still unclear (15). Treatment of FTC is based on total or near-total thyroidectomy, supplemented by radioiodine therapy after surgery. In high-risk patients, TSH suppression therapy and targeted agents (e.g., lenvatinib) may improve prognosis. Postoperative pathology indicated that the maximum diameter of the tumor was less than 4cm. After thorough communication with the patient and her family, they refused to have the remaining thyroid completely removed and opted for close observation and follow-up. The uniqueness of this case lies in the rarity of collision tumors of thyroid PEComa and follicular carcinoma, suggesting the need for clinical alertness to the possibility of coexistence of multiple primary tumors and the development of a precise treatment plan through multidisciplinary collaboration.

## Conclusion

In conclusion, we document the first case of a collision tumor comprising malignant PEComa and FTC in the thyroid. This case underscores the critical importance of comprehensive immunohistochemical profiling in accurately diagnosing complex thyroid neoplasms. It expands the spectrum of primary thyroid tumors and highlights that pathologists and clinicians should be aware of the possibility of collision tumors to guide appropriate therapy.

## Data Availability

The original contributions presented in the study are included in the article/supplementary material. Further inquiries can be directed to the corresponding author.
